# Pattern of Respiratory Diseases, Morbidities and Outcome in Patients Admitted in Respiratory Ward of a Tertiary Care Hospital: A Descriptive Cross-sectional Study

**DOI:** 10.31729/jnma.5613

**Published:** 2020-12-31

**Authors:** Pankaj Pant, Aishana Joshi, Bibek Man Shrestha, Babin Basnet, Niraj Bam, Santa Kumar Das

**Affiliations:** 1 Department of Pulmonology and Critical Care Institute of Medicine, Maharajgunj, Kathmandu, Nepal; 2 Department of General Practice and Emergency Medicine, Institute of Medicine, Maharajgunj, Kathmandu, Nepal; 3 Institute of Medicine, Maharajgunj, Kathmandu, Nepal

**Keywords:** *pattern*, *respiratory diseases*, *tertiary center*

## Abstract

**Introduction:**

Respiratory diseases are leading cause of morbidity and mortality worldwide imposing significant global health burden. The admission rate of patients is the indication of the overall workload in the ward. The aim of this study was to find the prevalence of admission of patients in the pulmonology ward among patients visiting pulmonology department of a tertiary care hospital.

**Methods:**

A descriptive cross-sectional study was conducted at Tribhuvan University Teaching Hospital. Medical records of all patients visiting pulmonology department and admitted in pulmonology ward from May 2018 to April 2020 were retrospectively reviewed. Data entry and analysis was done in SPSS version 20.0. Descriptive statistics were performed.

**Results:**

A total of 30,480 patients visited the pulmonology department in the two-year study period. Out of them, 1296 (4.25%) patients were admitted in the pulmonology ward. Eleven respiratory diseases were identified as primary causes for admission. Acute exacerbation of chronic obstructive pulmonary disease (44.5%), pneumonia (26.3%), tuberculosis (11%), lung cancer (5%) and bronchiectasis (3.9%) ranked top five causes for admission.

**Conclusions:**

Respiratory diseases impose tremendous burden in health care setting. Acute exacerbation of chronic obstructive pulmonary disease, pneumonia and tuberculosis remain an important cause of respiratory admissions in our study.

## INTRODUCTION

Respiratory diseases constitute an important global health burden. Acute respiratory infections, pneumonia, tuberculosis, obstructive and restrictive lung diseases, pleural diseases, and malignancies are common respiratory conditions for hospital admission.

Chronic obstructive pulmonary disease (COPD), lower respiratory tract infections, lung cancer and tuberculosis have been identified as top four respiratory diseases among ten leading causes of death worldwide.^1^ The forum of international respiratory societies (FIRS) estimated that 65 million people have moderate to severe COPD resulting in 3 million deaths per year, making it third leading cause of death worldwide.^2^ Currently, asthma affects an estimated 334 million people worldwide and is projected to increase to 400 million by the year 2025.^3^ In 2015, 10.4 million people developed TB with 1.4 million global deaths were reported.^4^

The aim of this study was to find the prevalence of admission of patients in the pulmonology ward among patients visiting pulmonology department of a tertiary care hospital.

## METHODS

This is a descriptive cross-sectional study conducted in the department of Pulmonology at Tribhuvan University Teaching Hospital (TUTH), a tertiary referral center in Kathmandu, Nepal. Ethical approval was obtained from Institutional Review Committee, TUTH (Reference no. 158/ (6-11) E2/076/077). All patients admitted in pulmonology ward from May 2018 to April 2020 were included in the study. Medical records of all patients admitted in pulmonology ward of TUTH over the period of two years were retrospectively reviewed and information on demographic data, pattern of respiratory diseases among admitted patients, co-morbidities, duration of hospital stay, and treatment outcome were retrieved. Treatment outcome was further categorized into discharge, intensive care unit (ICU) admission, leave against medical advice (LAMA) and mortality.

Data were extracted from in-hospital patient records. Data entry and analysis was done in Statistical Package for the Social Sciences (SPSS) version 20.0. Descriptive analysis was done.

## RESULTS

A total of 30,480 patients visited the pulmonology department in the two-year study period. Out of them, 1296 (4.25%) patients were admitted in the pulmonology ward. Eleven respiratory diseases were identified as primary causes for admission in respiratory ward. Acute exacerbation of chronic obstructive pulmonary disease (COPD) was the most common condition for admission followed by pneumonia and tuberculosis (TB) respectively (Table 1).

**Table 1:** Causes for admission in respiratory ward.

Primary diagnosis	n (%)
COPD exacerbation	577 (44.5)
Pneumonia	341 (26.3)
Tuberculosis	142 (11.0)
Lung cancer	65 (5.0)
Bronchiectasis	51 (3.9)
Pneumothorax	50 (3.9)
Interstitial lung disease	21 (1.6)
Empyema	19 (1.5)
Lung abscess	17 (1.3)
Acute severe asthma	11 (0.8)
Hydatid cyst	2 (0.2)
Total	1296 (100)

**Figure 1:**
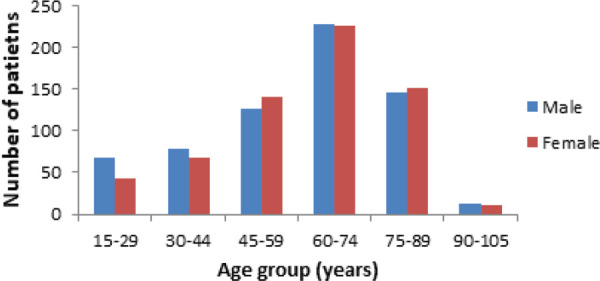
Age distribution of patients.

The mean age of the patients was 60.36±18.44 years with range from 16 to 103 years. Among the patients admitted, the highest number of patients was in age group 60-74 years in both sexes. The sex distribution of patients was similar in both sex with 659 (50.8%) male and 637 (49.2%) females (Figure 1).

**Figure 2:**
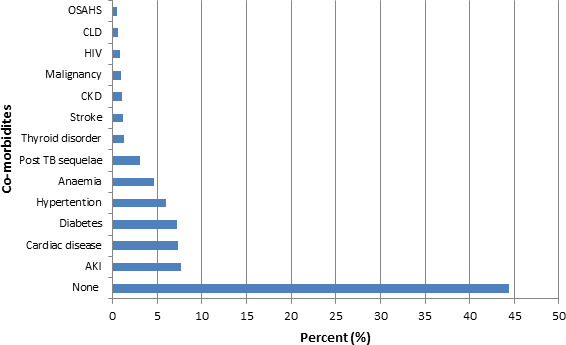
Co-morbidities in patients admitted with respiratory diseases.

Nearly 55% of patients had one of the 13 co-morbidities. Acute kidney injury (AKI) was the most common co-morbidity seen in 100 (7.7%) patients. Other common co-morbidities were cardiac disease in 95 (7.3%), diabetes mellitus in 93 (7.2%), hypertension in 78 (6%), anemia in 61 (4.7%) and post TB sequelae in 40 (3.1%) patients (Figure 2).

*OSAHS- Obstructive sleep apnea hypopnea syndrome, ϯCLD- Chronic liver disease, Ŧ HIV- Human immune deficiency virus, || CKD- Chronic kidney disease, ¶AKI- Acute kidney injury

The mean duration of hospital stay was found to be 8.8±6.18 days with range from 3 to 33 days. The highest duration of hospital stay was seen in patients with pneumothorax which was 11.12±7.51 days while lowest for acute severe asthma with duration of 5.62±2.50 days.

**Figure 3:**
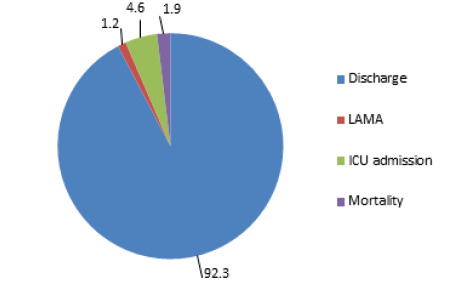
Treatment outcome of patients.

More than 90% of patients (n=1196) admitted in respiratory ward got discharged. Fifteen (1.2%) patients left against medical advice (LAMA), 60 (4.6%) patients deteriorated and required subsequent admission in intensive care unit (ICU) while 25 (1.9%) patients had in-hospital mortality. Out of total ICU admissions, the highest admission rate was 58.3% (n=35) seen in patients with acute COPD exacerbation. The highest mortality rate was also seen in COPD patients which accounted to 36% (n=9) of total in-hospital mortality (Figure 3).

## DISCUSSION

We found that 1296 patients with respiratory diseases were admitted in respiratory ward over two years. In a study done by Pokharel BR et al^5^ in a teaching hospital of Nepal, respiratory diseases (31.73%) were the most common cause among 1040 admissions in medical ward. The mean age of patients in respiratory ward was 60.36±18.44 years with range from 16 to 103 years. In a similar study done at a tertiary centre in India over a period of two years by Kumar A et al,^6^ the mean age for admission was found to be 48.6±12 years. Sachdeva R et al^7^ in a study done at a university hospital in India observed 50.64±15.71 years. We found a similar proportion of male and female patients in our study. There were 50.8% male and 49.2% female patients. In contrast to our study, Kumar A et al^6^ observed a male preponderance for admission with 70% males and 30% females out of 3337 patients who got admitted to pulmonary medicine ward. Higher proportion of male patients were also observed by Sachdeva R et al^7^ and also in a study done in Nigeria by Umoh VA et al^8^ which observed male to female ratio for admission to be 3.5:1 and 1.7:1 respectively. This discrepancy in sex distribution among the patients could be implicated to the genetic differences and health seeking behavior and practices among male and female towards lung disease. We found that 11 respiratory diseases were primary causes for admission in respiratory ward. COPD, pneumonia, tuberculosis, lung cancer and bronchiectasis were the top five causes of admission in respiratory ward while combined prevalence of other diseases like pneumothorax, interstitial lung disease, lung abscess, empyema, asthma and hydatid cyst comprised of less than 10% of total admissions.

Acute exacerbation of COPD was the most common respiratory condition seen in 44.5% patients followed by pneumonia in 26.3%, tuberculosis in 11%, lung cancer in 5% and bronchiectasis in 3.9% of patients. These findings are consistent with study done by Kumar A et al^6^ which found COPD (29.3%), TB (14.8%), pneumonia (9.3%), asthma (5.5%) and bronchiectasis (2.8%) as top five causes for admission in respiratory ward. Sachdeva R et al^7^ also reported tuberculosis, COPD, pneumonia, lung cancer and asthma as major causes for admission. Similar findings were reported in a study done by Alamoudi OS et al^9^ in Saudi Arabia and Umoh VA^8^ in Nigeria. Though the top five primary diseases for admission in respiratory ward in our and other studies were similar, there was striking variation in prevalence of these diseases among these studies. Another distinct contrasting feature in our study as compared to others is the lower prevalence of asthma as seen in 0.8% patients. The differences in occurrence of diseases could be implicated to diverse geographic and environmental variations among these regions.

The higher prevalence of COPD in our study could be related to the upsurge in national prevalence of COPD in recent years. In a study on prevalence of selected non-communicable diseases in Nepal conducted by the Nepal Health Research Council (NHRC), COPD had the highest prevalence (11.7%) among non-communicable diseases reported in the participants.^10^ Tobacco smoking, indoor and outdoor air pollution are major contributing factors for higher prevalence of COPD in Nepal. Biomass fuel is used widely used across Nepal for cooking purposes in more than 87% of households in rural areas as compared to 52% in urban areas.^11^ In 2016, the Global burden of disease study estimated the prevalence of COPD in Nepal as 4,810 per 1,00,000 which was accountable for 5.72% of all deaths.^12,13^

A myriad of co-morbidities were found to be associated with respiratory diseases. More than 55% of patients had one of the 13 co-morbidities among which acute kidney injury was the most common co-morbidity seen in 7.7% patients followed by cardiac disease in 7.3%. On contrary, Kumar A et al^6^ found hypertension and diabetes mellitus as the commonest co-morbidities which ranked third and fourth in our study. Umoh VA et al^8^ in Nigeria observed HIV/AIDS and hypertension as the commonest co-morbidities.

Pre-existing cardiac diseases was the second most common co-morbidity seen in our study. Concomitant respiratory and cardiac diseases result in overlapping symptoms of chest pain and dyspnoea in patients. Features of chronic cor pulmonale was seen in 29.3% of 577 COPD patients. A study done by Shrestha B et al^14^ in Nepal reported more than half of the patients presenting to hospital with COPD had features of chronic cor pulmonale as shown in echocardiography.

The mean duration of hospital stay was 8.8 ± 6.18 days with range from 3 to 33 days. The highest duration of hospital stay was seen in patients with pneumothorax (11.12 ± 7.51 days) while lowest in acute severe asthma (5.62 ± 2.50) days. Similar range for duration of hospital stay (2 to 28 days) was observed by Kumar A et al^6^ but they reported lower average duration of 5.2 ± 2.5 days than in our study. Sachdeva R et al^7^ also observed lower duration of hospital stay which was 6.91 ± 5.14 days while higher duration was observed by Umoh VA et al^8^ which was 15.4 ± 6.4 days but with similar range for duration (3 and 35 days) as observed in our study.

More than 90% of patients admitted in respiratory ward got discharged, 1.2% patients left against medical advice (LAMA), 4.6% required subsequent ICU admission while 1.9% patients had in-hospital mortality. Similar proportion of discharges were reported by Kumar A et al^6^ and Umoh VA et al^8^ but higher in-hospital mortality as compared to our study were reported in both of these studies.

## CONCLUSIONS

Respiratory diseases impose an immense burden in health care setting. COPD, pneumonia, tuberculosis, lung cancer and bronchiectasis remained top five causes of respiratory admissions in our study. This is a hospital-based study from a tertiary referral center in Nepal potentially serving as preliminary findings upon which future studies can be based.
